# Rebuttal to Correspondence on “Diffusion in Porous Rock Is Anomalous”

**DOI:** 10.1021/acs.est.5c07930

**Published:** 2025-07-02

**Authors:** Ashish Rajyaguru, Ralf Metzler, Ishai Dror, Daniel Grolimund, Brian Berkowitz

**Affiliations:** † Department of Earth and Planetary Sciences, 34976 Weizmann Institute of Science, Rehovot 7610001, Israel; ‡ Institute for Physics and Astronomy, 26583 University of Potsdam, 14476 Potsdam, Germany; § Asia Pacific Centre for Theoretical Physics, Pohang 37673, Republic of Korea; ∥ Paul Scherrer Institut, 5232 Villigen, Switzerland

Rajyaguru et al.^1^ investigated the diffusion behavior of an inert chemical tracer in porous rocks, using a specially designed experimental diffusion cell and a comparative quantification via models for Fickian and anomalous (non-Fickian) diffusion.

In their Correspondence on this paper, Chen and Li claim that our conclusion, namely, that the measured diffusion is anomalous, is “not reliable due to the use of an incorrect mathematical model”. We disagree with this claim and show that Chen and Li base their argument on a misrepresentation of the experimental design, the actual measurements, and their analysis.

Chen and Li present in their Figure 1 what they claim to be the experimental setup employed by Rajyaguru et al.^1^ However, this figure and the subsequent text clearly demonstrate that Chen and Li seriously misrepresent the experiments and analysis presented by Rajyaguru et al.^1^ and thus reach an incorrect conclusion regarding our paper.

Specifically, while Chen and Li recognize that Rajyaguru et al.^1^ examined tracer diffusion (not *gas* diffusion, as they erroneously state) in a fully water-saturated, semi-infinite porous rock domain, Chen and Li do not recognize that our principal measurements were *not* performed using the setup depicted in their Figure 1. In other words, we did *not* simply measure tracer concentrations in the inlet and outlet reservoirs and then apply a model for anomalous diffusion that assumes a semi-infinite (zero concentration) boundary condition at the outlet. Rather, our diffusion cell design, illustrated clearly in Figure 1 of Rajyaguru et al.^1^ (and reproduced here (Figure 1)), with the presence of the sampling slit and mimicking of a semi-infinite domain, is fundamentally distinct from that shown in Figure 1 of Chen and Li.

**Figure 1:**
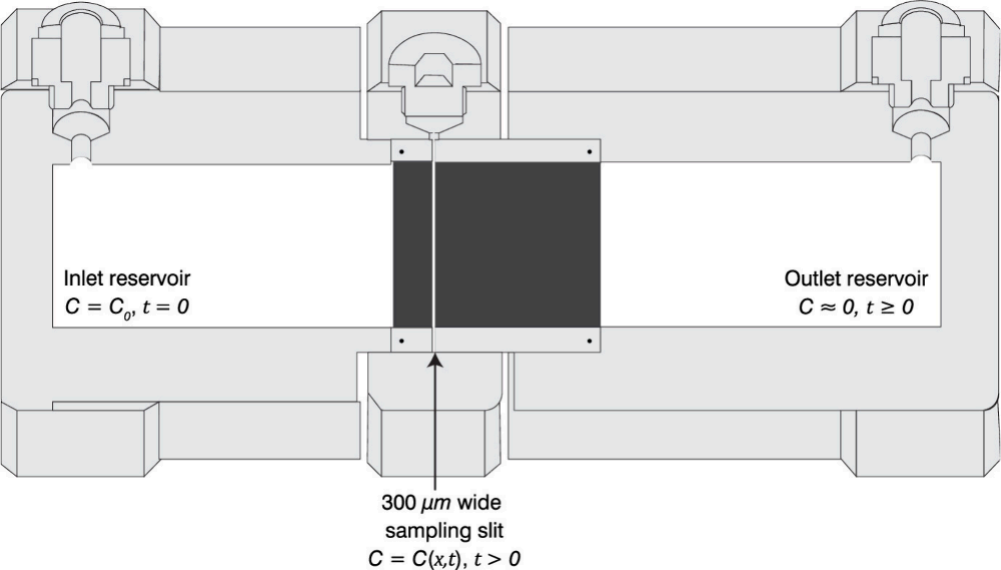
Schematic outline of the diffusion cell. Reproduced from ref 1. Copyright 2024 American Chemical Society.

As explained clearly in the Methods and Materials (Diffusion Cell Setup) of Rajyaguru et al.,^1^ the diffusion cell consisted of two reservoirs, sandwiching a cylindrical diameter rock core cut into two sections with lengths of 10 and 35 mm. The two rock samples were separated by a thin slit of 300 μm. This design enabled mimicking of tracer diffusion under a semi-infinite boundary condition over the duration of the experiments, namely, with a constant concentration inlet boundary (*C*(0, *t*) = *C*_0_), a zero concentration at the outlet boundary (functionally, *C*(∞, *t*) = 0), and the tracer breakthrough being measured in the sampling slit. Significantly, the longer (35 mm) rock core between the sampling slit and the outlet reservoir acts as a continuation of the (10 mm) rock core section of real interest, mimicking a single long (45 mm) sample. Measurements of the tracer concentration in the sampling slit represent the tracer concentration at a distance of 10 mm from the inlet reservoir, in a diffusion cell with an essentially semi-infinite boundary condition (pseudo-zero concentration an additional 35 mm downstream of the slit).

Rajyaguru et al.^1^ confirmed, at the conclusion of the experiments (which were 60–67 days in duration), that the changes in initial inlet and outlet reservoir concentrations were insignificant in the context of the analysis, so that analysis of the breakthrough curves measured in the slit was justifiably based on the semi-infinite conditions *C*(0, *t*) = *C*_0_ and *C*(∞, *t*) = 0. The tracer concentration will of course ultimately become uniform throughout the closed system diffusion cell at extremely long times, but this is irrelevant for the experimental setup, the experiment duration, the breakthrough curve measurements in the sampling slit, and the subsequent quantitative analysis provided by Rajyaguru et al.^1^ In other words, the amount of tracer arriving at the outlet reservoir within the experimental time is negligible.

Thus, the conclusion by Chen and Li that “the conclusion of anomalous diffusion in rocks presented by the authors is not reliable due to the use of an incorrect mathematical model” is completely unfounded and incorrect.
